# Impact of central complex lesions on innate and learnt visual navigation in ants

**DOI:** 10.1007/s00359-023-01613-1

**Published:** 2023-02-15

**Authors:** Cornelia Buehlmann, Scarlett Dell-Cronin, Angela Diyalagoda Pathirannahelage, Roman Goulard, Barbara Webb, Jeremy E. Niven, Paul Graham

**Affiliations:** 1 School of Life Sciences, University of Sussex, Brighton, BN1 9QG UK; 2 School of Informatics, University of Edinburgh, Edinburgh, EH8 9AB UK; 3 Lund Vision Group, Department of Biology, Lund University, 223 62 Lund, Sweden

**Keywords:** Central complex, Innate visual behaviour, Visual navigation, Wood ants, *Formica rufa*

## Abstract

Wood ants are excellent navigators, using a combination of innate and learnt navigational strategies to travel between their nest and feeding sites. Visual navigation in ants has been studied extensively, however, we have little direct evidence for the underlying neural mechanisms. Here, we perform lateralized mechanical lesions in the central complex (CX) of wood ants, a midline structure known to allow an insect to keep track of the direction of sensory cues relative to its own orientation and to control movement. We lesioned two groups of ants and observed their behaviour in an arena with a large visual landmark present. The first group of ants were naïve and when intact such ants show a clear innate attraction to the conspicuous landmark. The second group of ants were trained to aim to a food location to the side of the landmark. The general heading of naïve ants towards a visual cue was not altered by the lesions, but the heading of ants trained to a landmark adjacent food position was affected. Thus, CX lesions had a specific impact on learnt visual guidance. We also observed that lateralised lesions altered the fine details of turning with lesioned ants spending less time turning to the side ipsilateral of the lesion. The results confirm the role of the CX in turn control and highlight its important role in the implementation of learnt behaviours that rely on information from other brain regions.

**Supplementary Information:**

The online version contains supplementary material available at 10.1007/s00359-023-01613-1.

## Introduction

Ant foragers travel diligently back and forth between their nest and feeding sites using a combination of innate and learnt navigational strategies (Buehlmann et al. 2020b; Knaden and Graham 2016). Innate strategies such as path integration (Collett and Collett 2000; Mueller and Wehner 1988; Wehner and Srinivasan 2003), pheromone trails (Harrison et al. 1989), attraction to food odours (Buehlmann et al. 2014), and/or innate responses to visual cues (Buehlmann and Graham 2022; Collett 2010; Graham et al. 2003) are vital during exploration of novel territory. Innate responses shape the ants’ paths and thus ultimately facilitate the learning of visual cues required for establishing habitual foraging routes to stable feeding sites (Collett 2010; Graham et al. 2003; Heusser and Wehner 2002). Visual navigation in ants has been studied extensively at the behavioural level (reviewed in Collett et al. 2013; Graham and Philippides 2017; Wehner et al. 2014; Zeil 2012). Direct evidence for which circuits underly ant behaviour is sparse, although there is rapidly increasing information from other insects about neural areas that are hypothesised to have a role. Recent lesion studies in ants (Buehlmann et al. 2020c; Kamhi et al. 2020) demonstrate the need for the mushroom bodies (MBs), as associative learning centres, to learn the valence of views that can be used for navigation. However, other brain regions are likely involved in the transformation of MB outputs to steering behaviour and MBs do not appear to be required in innate visual orientation (Buehlmann et al. 2020c).

The central complex (CX), a collection of neuropils located at the midline of the insect brain (Strausfeld 1999), receives sensory input allowing an insect to keep track of the direction of sensory cues relative to its own orientation (Seelig and Jayaraman 2015), and to directly control movement (Martin et al. 2015). Studies have shown that the CX plays a role in insect orientation behaviours that use celestial information (locusts: (Pegel et al. 2018; Vitzthum et al. 2002); dung beetles: (el Jundi et al. 2015); monarch butterfly: (Heinze et al. 2013; Heinze and Reppert 2011); bees: (Stone et al. 2017); crickets: (Sakura et al. 2008); fruit flies: (Giraldo et al. 2018)), and terrestrial visual information (fruit flies: (Green et al. 2019; Kuntz et al. 2012; Liu et al. 2006; Neuser et al. 2008; Ofstad et al. 2011; Seelig and Jayaraman 2013); cockroaches: (Ritzmann et al. 2008); locust: (Rosner and Homberg 2013)). While fundamental knowledge has been gained from electrophysiological studies in which animals are restrained, we also have a handful of examples where neural recordings in the CX were performed in moving animals (e.g., Beetz et al. 2022; Green et al. 2019; Wosnitza et al. 2022). In insects, the CX has a role in the control of speed, turning behaviour, and spatial orientation (reviewed in Fisher 2022; Honkanen et al. 2019; Pfeiffer and Homberg 2014; Varga et al. 2017). Maintaining a stable direction relative to celestial or terrestrial visual cues allows insects to move in a straight line into a chosen direction but this ability needs to be augmented to account for the flexible visual navigation behaviours seen in ants and other species.

Many goal-directed behaviours executed by insects require associative learning, and the formation of long-term memories. This is particularly important for insects that engage in central place foraging, because they must learn the location of the nest and be able to return to it from multiple foraging sites. The CX has been implicated in visual place learning in fruit flies (Ofstad et al. 2011). Also, an increase of the CX volume was shown in new ant foragers that were exposed to skylight information during the learning of navigationally relevant information at the offset of their foraging career, indicating its involvement in navigation (Grob et al. 2017). A number of computational models have shown, by reproducing behavioural observations, that the CX circuit is a plausible substrate for innate and learnt visual navigation in ants (Goulard et al. 2021; Sun et al. 2020, 2021; Wystrach et al. 2020).

We show here direct evidence for the involvement of the CX in innate and learnt visual navigation of freely moving wood ants.

## Materials and methods

### Ants

Experiments were performed with laboratory kept wood ants *Formica rufa L.* collected from Ashdown Forest, East Sussex, UK. Ants were kept in the laboratory under a 12 h light: 12 h darkness cycle at 25–27 °C. Ants were fed ad libitum with sucrose, dead crickets and water. During the experiments, food was limited to increase the ants’ foraging motivation, but water was permanently available. All experiments were undertaken with ethical approval from the University of Sussex.

### Behavioural setup

**Fig. 1:**
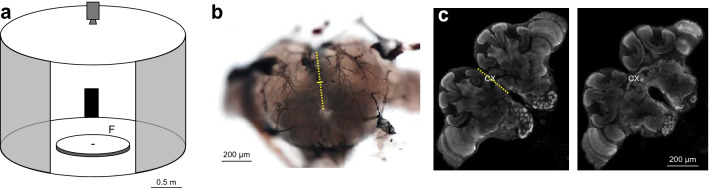
Behavioural setup and CX lesion locations. **a** Experimental arena in which naïve and trained ants were recorded. Circular white platform (radius: 60 cm) is located in the centre of a cylinder (radius: 1.5 m, height: 1.8 m). A 20° wide black rectangle (height: 90 cm, width: 52 cm) is mounted at the inner wall of the surrounding cylinder. Feeder (F) was placed at the edge of the white platform 30° to the right edge of the black rectangle. A camera recorded the ants’ paths from above. A small door permitted access to the arena shown here open and larger for clarity. **b** Mechanical lesions were made relative to landmarks (including tracheal branches) visible on the anterior surface of the brain. The bottom of the central tracheal branch, directly below the medial ocellus, was used as a superficial marker of the position of the CX. The bottom of it is approximately 53% down the midline of the brain, i.e., 218 µm down the midline that has a length of 409 µm in the brain shown. Yellow dashed line indicates brain midline. Small yellow line shows lesion locations, i.e., lesions were done approximately 20 microns to the left or right offset to the midline. **c** Confocal scan of an anti-synapsin labelled wood ant brain shown at two different heights. Lower edge of CX is around 58% down the midline (dotted line). CX width is approximately 200 µm (measured at max width)

The centre of the circular platform (120 cm in diameter) was placed within a cylindrical arena (diameter 3 m, height 1.8 m) with white walls (Fig. 1a; (Buehlmann et al. 2020c)). A 20° wide rectangle (height: 90 cm, width: 52 cm) was placed on the inner wall of the surrounding cylinder. To remove possible olfactory cues, the surface of the platform was covered with white paper, which was rotated between recordings. The centre of the platform consisted of a cylindrical release chamber of 6.5 cm diameter, which was remotely lowered to release the ant onto the platform. The ants’ position and body orientation were recorded every 20 ms using a tracking video camera (Trackit, SciTrackS GmbH).

To test the ants’ innate visual response, ants with no prior experience to this setup were released from the release chamber and their paths were recorded. To test the ants’ learnt visual response, other groups of ants underwent a training to learn to find a drop of sucrose on a microscope slide that, from the centre of the platform, was 30° to the right of the visual cue described above. During this training, individually marked ants were taken from the nests and released in the centre of the platform. Ants performed approximately 10 group training runs before being released and recorded individually. Once an ant had reached the feeder and started to feed, it was transferred into a feeding box and the next ant was released. Ants were considered to be reliable and accurate if they had approached the feeder directly on three consecutive training runs.

### Lesion procedures

We made lateralised lesions in the CX in the brains of naïve ants with no prior experience of this behavioural setup as well as in the CX of ants that have learnt to navigate to the feeder location. These ants were immobilised on ice for 90 s and harnessed in a custom-made holder keeping their head fixed with plasticine while their body was free to move. Antennae were restrained with a pin. To access the CX, a small window was cut with a piece of razor blade below the medial ocellus. Mechanical lesions were made relative to landmarks (including tracheal branches) visible on the anterior surface of the brain, however, we did not control the depth. The bottom of the central tracheal branch, directly below the medial ocellus, was used as a superficial marker of the position of the CX. The bottom of it is approximately 53% down the midline of the brain (i.e., 218 µm down the midline that has a length of 409 µm in the brain shown in Fig. 1b). Other measurements made using a confocal microscope confirmed that this is within the range of the CX, the lower edge of which is approximately 58% down this midline (Fig. 1c). We made lateral CX lesions approximately 20 µm to the left or right of the midline. The width of the CX is around 200 µm at its maximum (Fig. 1c). These measurements ensured that we lesioned the CX. However, we did not visualise the lesion locations after ants were tested. This prevents us from localising the lesions precisely. Glass capillaries used for the mechanical lesions (Harvard Apparatus, Cambourne, UK; 30-0035; 1.00 mm outer diameter, 0.78 mm inner diameter, 10 mm length) were pulled with a P-97 Micropipette Puller (Sutter Instrument, Novato, California, USA) and then broken manually to a tip size of 10 µm and dipped in black ink to aid visibility of the capillary during the lesioning. After inserting the capillary into either the left (LLes) or right (RLes) side of the CX, the cuticle lid was put back and secured in place with a small drop of cyanoacrylate adhesive. Control ants (Contr) were handled and dissected in the same way as lesioned ants but the glass capillary was not inserted. After the CX lesion (or control) was performed, ants were placed into a box and allowed to rest for 10 min before their navigational performance was recorded on the platform.

### Analysis

Paths were analysed in Matlab R2021b with custom written scripts. Trajectories were trimmed at *r* = 50 cm, where *r* is the distance from the platform centre, and the final heading directions determined at that point. Walking speed and path straightness (beeline/total path length) were calculated for trajectories from *r* = 3.25 cm to *r* = 50 cm. A more detailed analysis was performed for the ants’ turning behaviour. To determine turns, we took the angular velocity trace for each trajectory and smoothed it with a window of 50 frames to remove high frequency noise, we segregated this trace into turns by looking for zero crossings. Turns that were less than 0.2 s were not included in further analysis as these are often caused by periods of very low velocity or short straight path segments. Left and right turns were determined for each trajectory and duration and angle of those turns calculated. For each trajectory, we calculated the mean turn duration and mean turn angle for left and right turns, respectively. For both turn duration and angle, we then looked at turn balance by normalising the mean time or angle taken up by left turns (left mean/(left mean + right mean) for each ant.

## Results

### Overall path structure in naïve ants is not affected by CX lesions

**Fig. 2:**
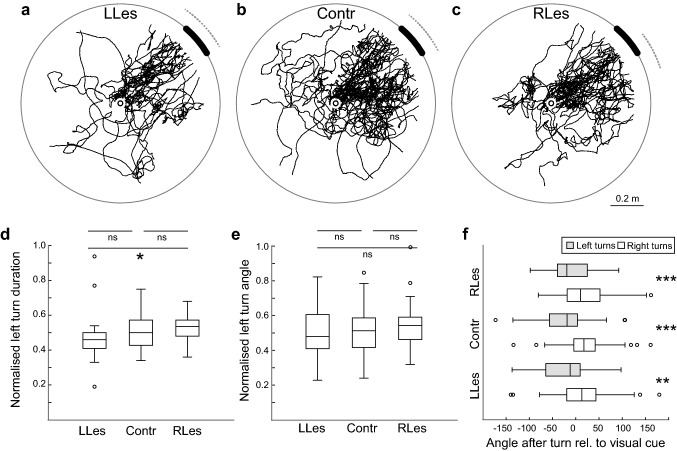
CX lesions do not affect innate visual attraction in wood ants but disrupt turning. **a** Paths of naïve ants lesioned on the left side of the CX (LLes) released at the centre of the arena are shown as black lines. Dotted arcs show 95% confidence intervals (CIs) of the heading directions. The visual cue is shown as a black bar at the platform edge instead of on the cylinder wall. **b** As for (**a**) but for control ants (Contr). **c** As for (**a**) but for ants with lesions on the right side of the CX (RLes). **d** Normalised left turn duration is significantly different in LLes and RLes ants (Kruskal Wallis with Dunn’s post hoc tests, *p* < 0.05). **e** Normalised left turn angle is not significantly different between the three groups (Kruskal Wallis with Dunn’s post hoc tests, all *p* > 0.05). **f** Mean angles at the end of right and left turns differed significantly from each other in all three conditions (Wilcoxon tests, all *p* < 0.01). Ants were oriented to the right relative to the centre of the visual cue at the end of a right turn, whereas ants were oriented to the left of the centre of the visual cue at the end of a left turn. Boxplots: median, 25th and 75th percentiles (edges of the boxes) and whiskers for extreme values not considered as outliers (circles). *ns* not significant; *p < 0.05; **p < 0.01; ***p < 0.001. For further statistics and sample sizes, see Results

CX lesions did not affect the ants’ innate preference for large dark objects, i.e., the innate visual orientation of CX-lesioned ants was not impaired (Fig. 2a–c). Control (Contr, *n* = 37 ants) and lesioned ants (left lesions, LLes, *n* = 24 ants; right lesion, RLes, *n* = 26 ants) were directed (Rayleigh test, all *p* < 0.001; Lengths of mean vector (*r*), Contr: 0.68, LLes: 0.75, RLes: 0.87). Ants from the three groups did not differ from each other in their path directions (Mardia–Watson Wheeler tests, all *p* > 0.05) and all three groups approached the visual cue, i.e., the 95% CI of the ants’ final heading direction encompassed the visual cue.

**Fig. 3:**
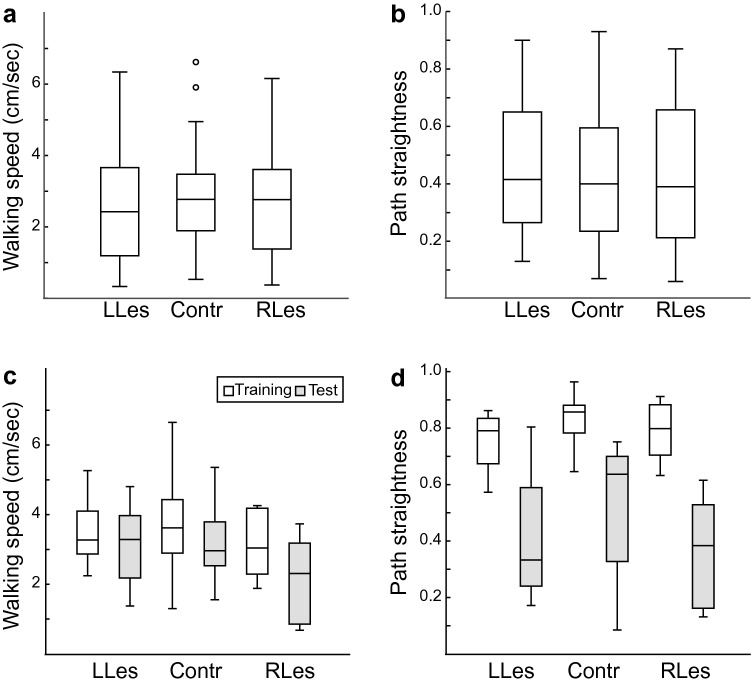
Walking speed and path straightness. **a** Walking speed did not differ between the control and the two lesioned groups in naïve ants (Kruskal Wallis with Dunn’s post hoc tests, all *p* > 0.05). **b** Path straightness was also not significantly different in these three groups of naïve ants (Kruskal Wallis with Dunn’s post hoc tests, all *p* > 0.05). **c** Walking speed did not differ between the training and test in all three groups (Wilcoxon tests, all *p* > 0.05). **d** In all three groups of trained ants, overall path straightness was significantly lower in tests than in trainings (Wilcoxon tests, all *p* < 0.05). Boxplots: median, 25th and 75th percentiles (edges of the boxes) and whiskers for extreme values not considered as outliers (circles)

Furthermore, walking speed (Fig. 3a) and overall path straightness (Fig. 3b) did not differ between the control and the two lesioned groups (Kruskal Wallis with Dunn’s post hoc tests, all *p* > 0.05). In all three groups, the number of left and right turns per trajectory did not differ from each other (Wilcoxon tests, all *p* > 0.05). Hence, lesions in the CX had no discernable impact on the ants’ ability to move and their overall path structure.

### CX lesions disrupt turning in innate visual orientation

Detailed path analysis revealed that the CX lesions disrupted the ants’ fine-scale turning behaviour. Duration and angle of all turns and means per ant for left and right turns, respectively, are shown in Online Resource 1. We analysed the turn balance by normalising the mean time taken up by left turns (left mean/(left mean + right mean) for each ant. We found that the two groups of lesioned ants were significantly different from each other (LLes vs. RLes, Kruskal Wallis with Dunn’s post hoc tests, *p* < 0.05). More specifically, lesioned ants had relatively shorter duration turns in the ipsilateral direction, i.e., LLes ants had relatively shorter turns to the left than RLes ants (Fig. 2d). The normalised left turn angles, however, were not significantly different between the three groups (Fig. 2e; Kruskal Wallis with Dunn’s post hoc tests, all *p* > 0.05), suggesting faster turns to the side ipsilateral to the lesion. As expected, we observed that the mean heading relative to the visual cue after a right and left turn differed significantly in all three conditions (Fig. 2f; Wilcoxon tests, all *p* < 0.01). At the end of a right turn, ants were oriented to the right relative to the centre of the visual cue, whereas at the end of a left turn, ants were oriented to the left of the centre of the visual cue.

### CX lesions disrupt visual navigation along a learnt route

**Fig. 4:**
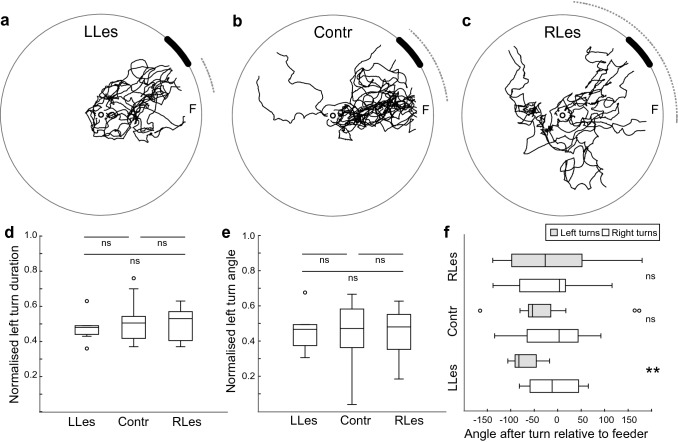
CX lesions disrupt learnt navigation. **a** Paths of trained ants lesioned on the left side of the CX (LLes) are shown as black line. **b** As for (**a**) but for control ants (Contr). **c** As for (**a**) but for ants with lesions on the right side of the CX (RLes). **d** Normalised left turn duration is not significantly different between the three groups (Kruskal Wallis with Dunn’s post hoc tests, all *p* > 0.05), but we see the same trend as in naïve ants with shorter durations for turns ipsilateral to the lesion. **e** The three groups do not differ from each other in normalised left turn angle (Kruskal Wallis with Dunn’s post hoc tests, all *p* > 0.05). **f** Mean angles after right and left turns differ in LLes ants (Wilcoxon test, *p* < 0.01) but not in RLes and Contr ants (Wilcoxon test, both *p* > 0.05). For more details of figure, see Fig. 2. For further statistics and sample sizes, see Results

Innate visual behaviours shape foraging and thus navigation but are not sufficient for efficient navigation and so we also tested the impact of CX lesions on the ability to navigate along a learnt route. Ants learnt to navigate to a feeder and once they were reliably navigating to the feeding site, we performed the CX lesion and tested the ants’ route knowledge afterwards. Paths in all three groups were still directed (Fig. 4a–c; Rayleigh test, all *p* < 0.05; Lengths of mean vector (*r*), Contr: 0.74, LLes: 0.97, RLes: 0.59). In the control group, the ants’ headings did not differ between the training and test (Contr, *n* = 14 ants; Mardia–Watson Wheeler test, *p* > 0.05), hence, these ants were still able to follow the learnt route. In CX-lesioned ants, however, the ants’ headings were significantly different in training and tests and ants no longer accurately approached the learnt feeder location (LLes, *n* = 9 ants; RLes, *n* = 9 ants; Mardia–Watson Wheeler test, both *p* < 0.01).

In all three groups, the walking speed did not differ between the training and test (Fig. 3c; Wilcoxon tests, all *p* > 0.05). Overall path straightness, however, was significantly lower in the tests than training runs in all three groups (Fig. 3d; Wilcoxon tests, all *p* < 0.05). The number of left and right turns, however, did not differ from each other in any of the three groups (Wilcoxon test, all *p* > 0.05).

Furthermore, we observe here the same trend for the normalised left turn duration as in naïve ants, however, this difference was not significant (Fig. 4d; Kruskal Wallis with Dunn’s post hoc tests, all *p* > 0.05). The three groups also did not differ from each other in the normalised left turn angles (Fig. 4e; Kruskal Wallis with Dunn’s post hoc tests, all *p* > 0.05). See Online Resource 2 for duration and angle of all turns and means per ant for left and right turns, respectively. In trained ants, we see a significant difference between the mean angles after a right and left turn in the LLes group (Fig. 4f; Wilcoxon test, *p* < 0.01) but not in the RLes and Contr groups (Wilcoxon test, both *p* > 0.05), but given the increase in sinuosity and small sample size these results seem broadly consistent with the results from naive ants. Altogether, we show here that CX-lesioned ants are no longer able to navigate towards a learnt feeder location.

## Discussion

In recent years, the CX has received attention as a brain area crucial for orientation in insects (Fisher 2022; Honkanen et al. 2019; Pfeiffer 2022; Pfeiffer and Homberg 2014; Turner-Evans and Jayaraman 2016; Varga et al. 2017). The CX receives pre-processed sensory input that allows insects to orient themselves relative to external cues (Seelig and Jayaraman 2015). Furthermore, the CX can directly control movement (Bender et al. 2010). Keeping a stable direction relative to celestial or terrestrial visual cues is, however, not always sufficient for navigation and many goal-directed behaviours require associative learning, and the formation of long-term memories. We show here that CX lesions have an impact on innate and learnt visual navigation (Figs. 2 and 4). More specifically, we show here that lesions in the CX lead to a lateralised disruption in ants’ turning behaviour but that naïve ants with lateralised CX lesions can still control their overall orientation towards the visual cue (Fig. 2). Ants that learnt to navigate along a visually guided route, however, are no longer able to accurately approach the learnt feeder location when the CX is lesioned (Fig. 4).

### Involvement of the CX in learnt visual navigation

Perhaps, the most striking result here is that lateralised CX lesions lead to a strong impact on learnt visual guidance. These ants reverted partially but not fully towards their innate visual response when the CX was lesioned. 

A possible interpretation of our result is that the CX is the basis of visual memory (Ofstad et al. 2011) but we have previously observed that such visual guidance is disturbed by MB lesions that leave the CX intact (Buehlmann et al. 2020c; Kamhi et al. 2020). We, therefore, conclude that the loss in CX-lesioned ants reflects an inability to use this memory to drive steering. This seems to confirm that MBs are a structure that encodes the valence of sensory inputs rather than encoding any explicit spatial information directly (Collett and Collett 2018; Matheson et al. 2022; Sun et al. 2020) and that the influence of MB on navigation could be completely dependent on the way that the CX encodes spatial information and drives steering (Goulard et al. 2021).

Tangential neurons in the fan-shaped body of the CX have input neuron dendrites that are found in brain areas that are also innervated by MB output neurons (Heinze et al. 2013; Matheson et al. 2022; Young and Armstrong 2010). These MB output neurons encode the valence of sensory cues (Aso et al. 2014), hence, if a current view matches with a learnt view, the current head-direction activity pattern in the proto-cerebral-bridge region of the CX could be imprinted onto the orientation coding neurons (Honkanen et al. 2019). Consistent with this idea, we know from recent connectomic work on *Drosophila* that approximately half of the MB output neurons directly synapse onto tangential neurons of the fan-shaped body (Hulse et al. 2021; Li et al. 2020). Our results are consistent with the assumption that our lesions have disturbed this pathway, preventing the MB signal from influencing the steering circuit to drive approach in the learned direction.

### Control of innate visual behaviour in the CX

Many insects show innate preferences, i.e., they perform fixed motor behaviours in response to specific visual stimuli. Tall objects are attractive for many insects, including ants (fruit flies: Gotz 1987; Strauss and Pichler 1998; Wehner 1972), locusts: (Wallace 1962), ladybirds: (Collett 1988), mantids: (Poteser and Kral 1995), ants: (Buehlmann and Graham 2022; Voss 1967), monarch butterflies: (Franzke et al. 2022)). Experiments in *Drosophila* have revealed that the CX is required for innate visual approaches (Bausenwein et al. 1994) and spatial learning paradigms in *Drosophila* revealed its importance for visual and spatial memory and directional decision making (Kuntz et al. 2012; Neuser et al. 2008; Ofstad et al. 2011; Seelig and Jayaraman 2013, 2015). Our lesion method allows us to perform lateralised lesions in the CX, however, we cannot get more detailed information about which part exactly was lesioned. Ring neurons in the ellipsoid body map visual cues spatially (Seelig and Jayaraman 2013) and computational simulations have revealed that the lateralised ellipsoid body input in *Drosophila* is suitable for innate responses to bar-like objects (Dewar et al. 2017; Wystrach et al. 2014). In addition, ellipsoid body neurones have been shown to be important for short-term spatial memory, during which they encode the position of a target (e.g., an attractive visual cue) and can guide movement towards it (Neuser et al. 2008) even when temporarily invisible. Given its conserved structure across insects, it is likely that the ellipsoid body plays a similar role in ants. With lateralised lesions, ants retained their innate attraction to the visual cue, suggesting that the wide field visual inputs from each visual hemisphere are sufficient for overall path control.

### Control of turning behaviour by the CX

The CX is also involved in fine-scale control of steering including the control of walking speed (Bender et al. 2010; Martin et al. 2015) and turning (Guo and Ritzmann 2013). The symmetrical features of the CX along the medial–lateral axis might suggest a lateralized role of the CX (see e.g., (Pfeiffer and Homberg 2014)). Our data suggest that ants had slightly faster turns in the ipsilateral direction, given that the mean duration of their left turns was shorter, while the angle turned was the same (Figs. 2 and 4). Faster movements have previously been described in ants that were not currently guided by visual information (Buehlmann et al. 2020a, 2018), hence, it could be that the faster turns are representative of reduced visual input during the control of ipsilateral turns in lesioned ants. Furthermore, Ridgel and co-workers showed in cockroaches that off-centre CX lesions resulted in lateralized navigational deficits (Ridgel et al. 2007). More specifically, lesioned cockroaches lost the ability to turn in the ipsilateral direction relative to the lesion (Guo and Ritzmann 2013; Ridgel et al. 2007). Furthermore, CX stimulation experiments showed evoked turning to the ipsilateral side, i.e., stimulating the left CX evoked left turns and vice versa (Guo and Ritzmann 2013; Martin et al. 2015). Our results in ants are consistent with the CX controlling steering in a lateralized way as shown in cockroaches.

It has previously been suggested that visual control of orientation in wood ants involves ‘correction points’ that are synchronised with the underlying path ‘wiggle’ or sinuosity (Collett et al. 2014; Lent et al. 2010). This suggests that there may be some independence between the control of underlying path sinuosity (perhaps from the lateral accessory lobes; (Clement et al. 2022; Steinbeck et al. 2020)) and of visual corrections. Lateralised lesions in ants might not, therefore, knock-out the control of all turns in one direction but might diminish the visual control of those turns. However, redundancy in the visual inputs that allow for innate visual orientation, mean that ants’ overall paths are still directed to the visual cue.

## Supplementary Information

Below is the link to the electronic supplementary material.

Supplementary file1 (PDF 195 KB)

Supplementary file2 (PDF 174 KB)

## Data Availability

The dataset generated during this study is available from the University of Sussex research repository (hosted by Figshare): 10.25377/sussex.22012652.
