# Supplementary material for: Impact of central complex lesions on innate and learnt visual navigation in ants
Source: J Comp Physiol A Neuroethol Sens Neural Behav Physiol. 2023 Feb 15;209(4):737–46. doi: 10.1007/s00359-023-01613-1 (PMC10354120; doi:10.1007/s00359-023-01613-1)
Supplement: Supplementary file 1 — Supplementary file1 (PDF 195 KB) [file 359_2023_1613_MOESM1_ESM.pdf]

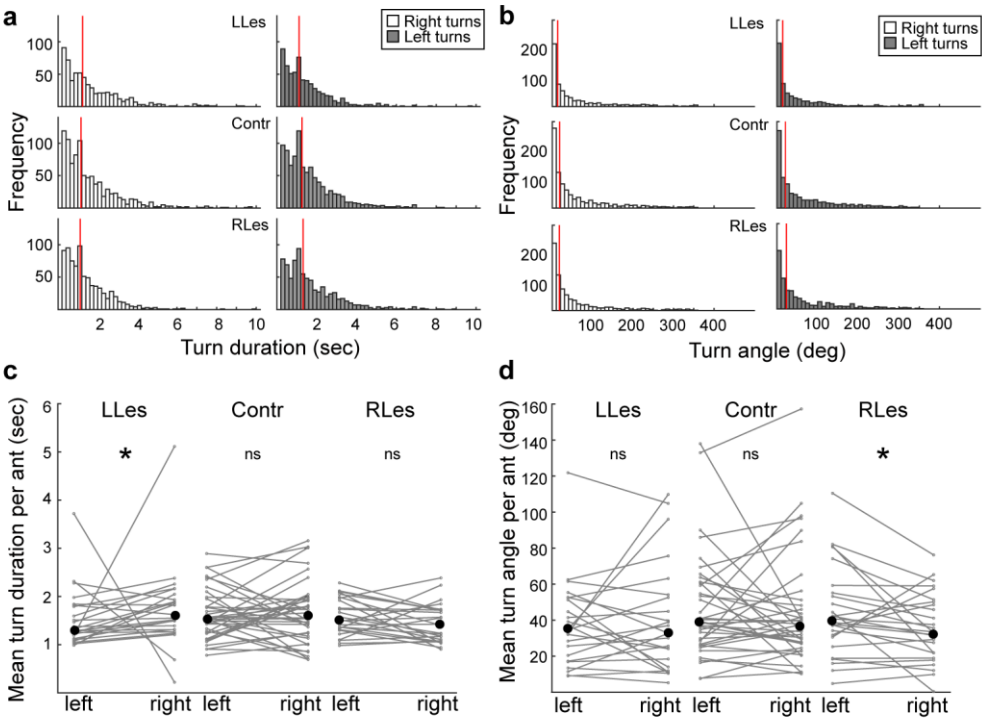

Online Resource 1: Turn durations and turn angles of naïve ants. (a) All turn durations of left and right turns are shown for LLes, Contr and RLes ants. Red line: median. Bin size: 0.2 sec. **(b)** All turn angles are shown for LLes, Contr and RLes ants. Red line: median. Bin size: 10°. **(c)** Mean turn duration per ant for left and right turns. Lines connect the left and right turn data points for one ant. Black circle: median. Wilcoxon test: ns, not significant; *, $p < 0.05$. **(d)** As in (c) but for mean turn angle per ant for left and right turns.
